# Association between days for concussion recovery and initial specialty clinic evaluation within 48 hours

**DOI:** 10.1186/s13102-024-00866-w

**Published:** 2024-04-02

**Authors:** Abel S. Mathew, Todd Caze, August M. Price, Desi Vasquez, John P. Abt, Scott O. Burkhart

**Affiliations:** 1 Children’s Health Andrews Institute for Orthopedics and Sports Medicine, Plano, TX USA; 2 Caze Concussion Institute, Omaha, NE USA; 3 Bellapianta Orthopaedics and Sports Medicine, Montclair, NJ USA; 4 Texas A&M International University, Laredo, TX USA; 5 University of Texas Southwestern—Psychiatry, Dallas, TX USA; 6 Present Address: Children’s Health Andrews Institute for Orthopaedics and Sports Medicine, 7211 Preston Rd., Plano, TX 75024 USA

**Keywords:** Concussion, Pediatric, Sports-related concussion, TBI, mTBI, Protracted recovery

## Abstract

**Background:**

Researchers have highlighted the importance of early access to concussion care within one week of injury in reducing recovery times. However, a persisting question for concussion researchers is “just how early is important?” The purpose of this study was to examine differences in recovery time as predicted by the number of days elapsed since injury (DSI) to initial evaluation among patients who had access to a specialty concussion clinic within seven days. We hypothesized that DSI group membership, even within seven days, would significantly predict risk of protracted recovery (i.e., beyond 21 days).

**Methods:**

In this archival study, retrospective data were gathered from electronic medical records between September 2020 to March 2022. Records of participants between ages 12–18, those diagnosed with a sports-related concussion based on initial clinic visit diagnosis by a medical provider and those who established care within seven days of injury at a large pediatric specialty concussion clinic were examined. Participants were divided into three DSI groups (patients seen in < 48 h: “acute”, patients seen between 49 h < and < 96 h: “sub-acute”, and patients seen between 97 < and < 168 h: “post-acute”). A general linear model was constructed to examine relationships between relevant concussion factors (e.g., Post Concussion Scale Score, neurodevelopmental history, psychiatric history, concussion history, migraine history, overall VOMS change score, cognitive testing, sex, age, race, and ethnicity) that were either significant in the preliminary analysis or in clinical judgement and recovery time. Adjusted odds ratios (OR) were derived from a binary logistic regression model, in which recovery time was normal (≤ 21 recovery days) or protracted (> 21 recovery days).

**Results:**

A total of 856 participants were eligible. Adolescents in the acute group (M = 15.12, SD = 8.04) had shorter recovery times in days compared to those in the sub-acute (M = 17.98, SD = 10.18) and post-acute (M = 21.12, SD = 10.12; *F* = 26.00, *p* < .001) groups. Further, participants in the acute (OR = 4.16) and sub-acute (OR = 1.37) groups who accessed specialty concussion clinics within 48 h were 4 times more likely to have a normal recovery and recovered approximately 6 days faster than the post-acute care group.

**Conclusions:**

Earlier concussion care access predicted recovery times and was associated with lower risk for protracted recovery.

## Background

The incidence rates of pediatric concussion is increasing, with current estimates ranging from 1.1 to 1.9 million injuries and costs to the economy exceeding $15 M annually [1]. Management of pediatric sports-related concussion (SRC) presents a clinical challenge as symptoms tend to evolve over the course of the recovery period, with a normal recovery window of two to three weeks [2]. While many variables influence pediatric and adolescent concussion recovery rates including prior psychiatric diagnosis [3–5], initial concussion symptom severity [6–8], headache/migraine history [9–11], concussion history [12–15], sex [16–20], and neurodevelopmental factors (ADHD, learning disability) [21, 22], only recently have researchers examined time to clinic as a critical factor in reducing the risk of protracted recovery [23, 24].

Recent research has shown that individuals who sought specialty concussion care within seven days improved faster than those who received care after seven days [23, 24]. Patients who delayed clinical care were six times more likely to experience protracted recovery [24], which is associated with continuing cognitive difficulties [25, 26], higher levels of psychological distress [27–30], sleep disruption [31–33], and numerous physiological symptoms [6, 32–36]. Additionally, continuing play, without evaluation and treatment, has been consistently linked to higher risk of longer recovery times in contrast to those who immediately report and seek care by a concussion specialist [37, 38]. Indeed, Charek et al., 2020 compared individuals with concussion who (1) were immediately removed from play, (2) continued to play for 15 min or less, or (3) continued to play for more than 15 min. Those who continued to play were between 5 to 11 times more likely to experience protracted recovery [39]. Additionally, in their study focusing on patients aged 5 to 9 years, Trbovich and colleagues (2024) found that their model accurately predicted 78% of patients with protracted recovery identifying the Vestibular/Ocular Motor Screen-Child (VOMS-C) score and days between concussion to first clinic visit as the most significant predictors of recovery duration [40].

Despite the growing evidence regarding time to clinic and its association with lower incidence of protracted recovery, generally, there is a paucity of research evaluating the effects of delayed care on SRC recovery outcomes within and outside the seven-day mark. In this study, we sought to examine differences in recovery time among adolescents who had access to specialty concussion care within seven days as there is currently no established literature evaluating differences in recovery among adolescents who receive SRC care within seven days. As such, we divided participants into three groups defined by days since injury (DSI; ≤ 48 h: acute, > 48 h and ≤ 96 h: sub-acute, and > 96 and < 168 h: post-acute). Considering our sample and the approximately equal increments of time within the seven-day window, it was hypothesized that adolescents in the acute DSI group would recover faster than those in the sub-acute and post-acute DSI groups from SRC. Further, it was hypothesized that DSI group membership would be most predictive of recovery time compared to other variables associated with concussion recovery (initial symptom severity, age, sex, psychiatric diagnosis, neurodevelopmental disorder, history of concussion, overall VOMS change score, cognitive testing).

## Methods

### Participants

This study involved a retrospective chart review of 1,159 patients evaluated for an SRC September 2020-March 2022 at a large pediatric specialty concussion clinic. Inclusion criteria were as follows: ages 12–18 years, diagnosed with SRC based on CISG 2017 criteria [41], established care within a specialty concussion clinic within seven days, and received medical clearance from the same specialty concussion clinic. In our study, recovery was defined as medical clearance to complete the return to play process. Exclusion criteria included cervicogenic injuries, and congenital or acquired neurological disorders not related to concussion. Patients were referred by emergency departments, athletic trainers, coaches, or self-referral. To increase generalizability of our sample, neurodevelopmental disorders (attention deficit hyperactivity disorder, ADHD, autism spectrum disorder, learning disability), psychiatric diagnoses (depression, anxiety), and individuals with migraines and previous concussion history were all included in our study. This study was approved by the local institutional review board (STU-2021–0334).

### Procedure

Of the 1,159 patients, 856 eligible participants were categorized into acute, sub-acute, and post-acute DSI groups. Date of injury was reported directly by the patient based on day of head impact or confirmed by referral source (e.g., athletic trainer, primary care provider, emergency department). All participants were evaluated by clinical staff (sports physician, neuropsychologist, or nurse practitioner) on the date of their initial visit to confirm or establish diagnosis of concussion, and follow-up visits to determine medical clearance. A positive concussion diagnosis was based on clinical history, Post Concussion Symptom Scale score (PCSS) [42], Vestibular Ocular Motor Screening (VOMS) [43], King-Devick test score [44], and brief computerized neuropsychological testing via C3 Logix [45]. Treatment included return-to-learn accommodations, return-to-play protocol implementation, and an individualized rehabilitation program (home exercises and physical therapy) based on PCSS score and clinical evaluation. Data collected from the electronic medical record included concussion diagnosis, age, sex, injury date, date of concussion evaluation, total number of visits, clinical characteristics (e.g., neurodevelopmental disorder, psychiatric diagnosis, history of migraines, history of concussion, PCSS total score), and date of medical clearance.

### Data analysis

To establish preliminary group statistical equivalence on potentially confounding risk factors for protracted recovery, we conducted Pearson’s chi-squared tests between categorical variables and one-way ANOVA to test for mean differences between groups, including whether there were significant differences in recovery time due to clinical factors (e.g., gender, concussion history) potentially affecting concussion recovery time. Pearson’s correlational analysis was conducted to evaluate the relationship between recovery time and continuous variables including age and PCSS total. A general linear model was then constructed examining the relationships between recovery time and its prediction by clinically relevant factors including psychiatric diagnosis, history of concussion, migraine diagnosis, neurodevelopmental diagnosis, sex, DSI group membership, race and ethnicity, overall VOMS change score (calculated using Elbin et al., 2018 criteria) [46, 47], average K-D test scores, C3 Logix cognitive testing (i.e., Trails A subtracted from Trails B to better account for cognitive flexibility components), age, and PCSS total. Interaction effects between categorical variables were initially examined but as they were statistically non-significant only main effects are presented for simpler interpretation.

Adjusted odds ratios (OR) for protracted recovery were derived from a logistic regression model, in which the outcome was normal (≤ 21 days) or protracted (> 21 days) recovery [37–39, 48, 49]. Individual predictors were systematically removed from the model if they did not significantly contribute to model performance (*p* < 0.05). Post hoc model diagnostics were conducted to evaluate the model for any violated assumptions. Statistical significance was set a priori at *p* < 0.05. Analyses were conducted using SPSS 28 software [50]. Post-hoc (multiple comparison) analyses included Ryan-Einot-Gabriel-Welsch F and Games-Howell tests, both assuming and not assuming equal variance among DSI groups [51, 52].

## Results

### Demographic and clinical characteristics

Participant demographic information and clinical characteristics can be found in Tables 1 and 2. Results from crosstabulation (chi-square) analyses indicated that all three DSI groups were similar in frequency of group members reporting factors potentially affecting concussion outcomes, including race and ethnicity, sex, and reported history of concussions, neurodevelopmental disorders, psychiatric disorders, and migraines (all χ^2^s > 0.05; Table 1). Groups were significantly different in terms of protracted recovery such that individuals in the post-acute group had the greatest number of individuals with protracted recovery followed by the sub-acute and acute groups, respectively (χ^2^s = 18.42; *p* < 0.001). There were significant differences between groups in terms of age (*p* = 0.02) and recovery days (*p* < 0.001), but no significant differences in total visits, PCSS total, overall VOMS change score, average K-D test scores, or cognitive testing (*p* > 0.05; Table 2). Post-hoc analyses showed that the acute and sub-acute groups differed by age (*p* = 0.02), while the post-acute group did not significantly differ by either group (*p* > 0.05). Recovery days were significantly different between all three groups (*p* < 0.001). Patients who were female, had a history of concussion, had a psychiatric diagnosis, older, and had higher PCSS scores, showed longer mean recovery times (*p* < 0.05; Tables 3 and 4). There were no differences in recovery time based on race and ethnicity, neurodevelopmental diagnosis, or history of migraines (Table 3).

**Table 1:** Chi-square analysis comparing DSI groups by demographic and clinical factor

	Total	Acute	Sub-acute	Post-acute	*X*^*2*^	*p*
Race/Ethnicity					17.59	0.13
White	463	136	193	134		
Black	73	23	25	25		
Asian	15	5	4	6		
Native Hawaiian/Pacific Islander	1	0	1	0		
Multiracial	25	7	7	11		
Unknown	166	54	58	54		
Hispanic	113	24	38	51		
Sex					5.66	0.06
Male	473	149	184	140		
Female	383	100	142	141		
Concussion history					1.67	0.43
Yes	279	74	114	91		
No	572	173	211	188		
Neurodevelopmental diagnosis					0.27	0.88
Yes	148	41	56	51		
No	708	208	270	230		
Psychiatric diagnosis					0.14	0.93
Yes	102	31	39	32		
No	754	218	287	249		
Migraine history					2.29	0.32
Yes	42	16	12	14		
No	814	233	314	267		
Protracted recovery						
Yes	110	15	43	52	18.42	< 0.001
No	746	234	283	229		

*n* = 856

^*^values significant at *p* < 0.05

**Table 2:** One-way ANOVA comparing DSI groups by mean age, recovery days, and PCSS total

	Total	Acute	Sub-acute	Post-acute	*F*	*p*
Age	14.91 (1.65)	14.66 (1.63)	15.04 (1.64)	14.98 (1.66)	4.06	0.02*
N	856	249	326	281		
Recovery days	18.18 (9.86)	15.12 (8.04)	17.98 (10.18)	21.12 (10.12)	25.99	< 0.001***
N	856	249	326	281		
Total visits	2.33	2.31 (0.52)	2.34 (0.59)	2.33 (0.56)	0.21	0.81
N	0.56	249	326	281		
PCSS total	29.53 (23.05)	30.08 (22.37)	28.09 (21.48)	30.68 (25.26)	0.95	0.39
N	775	229	290	256		
Overall VOMS	16.13 (16.16)	15.10 (16.27)	16.62 (15.94)	16.45 (16.16)	0.71	0.49
N	853	249	324	280		
Average K-D test	20.30 (6.70)	20.65 (6.68)	19.93 (7.13)	20.40 (6.19)	0.81	0.44
N	796	237	298	261		
Trails B-A	21.75 (15.68)	22.55 (16.76)	21.66 (13.88)	21.74 (15.70)	0.25	0.78
N	823	242	311	270		

*n* = 856

^*^values significant at *p* < 0.05

^**^values significant at *p* < 0.01

^***^values significant at *p* < 0.001

**Table 3:** Descriptive statistics and F-test results for demographic and clinical factors comparing mean recovery times in days

	Mean recovery time in days	*SD*	*F*	*p*
Race/Ethnicity			0.93	0.47
White	18.21	9.94		
Black	18.49	10.9		
Asian	20.73	9.38		
Native Hawaiian/Pacific Islander	7	-		
Multiracial	16.2	9.36		
Unknown	17.4	9.25		
Hispanic	19.19	9.9		
Sex			27.1	< 0.001***
Male	16.63	9.08		
Female	20.1	10.44		
Concussion history			13.6	< 0.001***
Yes	19.94	10.78		
No	17.3	9.26		
Neurodevelopmental diagnosis			0.15	0.7
Yes	18.47	9.15		
No	18.12	10.01		
Psychiatric diagnosis			5.65	0.02*
Yes	20.35	10.14		
No	17.89	9.79		
Migraine history			0.68	0.41
Yes	18.43	8.27		
No	19.7	9.77		

*n* = 856

^*^values significant at *p* < 0.05

^**^values significant at *p* < 0.01

^***^values significant at *p* < 0.001

**Table 4:** Pearson correlation between age, PCSS Total, and recovery time in days

Measure	1	2	3	4	5	6
1. Age	1	0.07*	0.06	0.07*	-0.14**	-0.10**
2. Recovery days		1	0.37**	0.26**	0.22**	-0.03
3. PCSS total			1	0.43**	0.37**	-0.08*
4. Overall VOMS				1	0.26**	-0.05
5. Average K-D test					1	-0.07
6. Trails B-A						1

*n* = 856

^*^values significant at *p* < 0.05

^**^values significant at *p* < 0.01

^***^values significant at *p* < 0.001

### DSI group membership and recovery days

There were significant differences in recovery times based on DSI group membership (*F* = 25.99, *p* < 0.001), with small to nearly medium effect sizes (see Table 5). Participants in the acute group had shorter recovery times in days (15.12 ± 8.04) than those in the sub-acute (17.98 ± 10.18) and post-acute groups (21.12 ± 10.12; Table 3).

**Table 5:** Results from a general linear model (GLM) regression analysis examining the relationships between clinical factors with recovery time in days

Predictor variable	*B*	SE	*p-*value	95% CI	*ηp2*
No psychiatric diagnosis	0.5	1.07	0.65	-1.61—2.60	< 0.001
No history of concussion	-1.97	0.77	0.01**	-3.48—-0.45	0.01
No history of migraine	1.88	1.52	0.22	-1.10—4.86	0.002
No neurodevelopmental diagnosis	0.75	0.93	0.42	-1.08—2.57	0.001
Sex—Male	-1.49	0.72	0.04*	-2.90—-0.08	0.007
DSI group 1	-3.99	0.9	< 0.001***	-5.76—-2.22	0.03
DSI group 2	-1.49	0.82	0.07	-3.10—0.12	0.005
DSI group 3	0^a^				
Race					
White	1.14	0.93	0.22	-0.68—2.97	0.002
Black	1.51	1.48	0.31	-1.39—4.42	0.002
Asian	1.45	2.78	0.6	-4.02—6.92	< 0.001
Native Hawaiian/Pacific Islander	-	-	-	-	-
Multiracial/Other	0.36	2.13	0.87	-3.82—4.54	< 0.001
Unknown	0^a^				
Ethnicity—Hispanic	1.43	1.22	0.24	-0.97—3.83	0.002
Average K-D test	0.13	0.06	0.02*	0.02—0.24	0.008
Trails B-A	0.002	0.02	0.94	-0.04—0.05	< 0.001
Overall VOMS change score	0.04	0.02	0.08	-0.01—0.09	0.005
Age	-0.11	0.23	0.62	-0.56—0.34	< 0.001
PCSS total	0.12	0.02	< 0.001***	0.08—0.16	0.06

a = this parameter is set to zero because it is redundant, *R*^*2*^ = 0.095 (Adjusted *R*^*2*^ = 0.089)

Native Hawaiian/Pacific Islander was not included in the parameter estimates as there was only 1 individual

*n* = 856

^a^reference category

^*^values significant at *p* < 0.05

^**^values significant at *p* < 0.01

^***^values significant at *p* < 0.001

### Clinical predictors of protracted recovery

The general linear regression model demonstrated that history of concussion, sex, DSI group membership, average K-D test scores, and PCSS total significantly predicted recovery days (see Table 5). When including these significant variables in a model predicting protracted recovery, the logistic regression analysis demonstrated that participants in the acute group, when compared to the post-acute group (OR = 4.16) and those without a concussion history (OR = 2.06), were significantly more likely to have normal recovery (see Table 6). Participants with higher PCSS scores were less likely to experience normal recovery (OR = 0.97). Overall, the model was significant, Nagelkerke Pseudo *R*^2^ = 0.18, *p* < 0.001.

**Table 6:** Logistic regression of clinical factors on likelihood of protracted recovery

Predictor variable	*B*	SE	*Relative risk odds ratio*
			Exp (B)
			(95% CI)
PCSS total	-0.03***	0.005	0.97 (0.96—0.98)
Average K-D test	-0.02	0.02	0.98 (0.95—1.01)
No history of concussion	0.72**	0.24	2.06 (1.28—3.29)
Sex—Male	0.24	0.24	1.27 (0.79—2.04)
DSI group 1	1.43***	0.35	4.16 (2.08—8.33)
DSI group 2	-0.31	0.26	1.37 (0.82—2.28)
DSI group 3	0^a^	–	–

Nagelkerke Pseudo *R*^*2*^ = 0.18

History of psychiatric diagnosis was removed from model in backward stepwise logistic regression due to non-significance

*n* = 856

^a^reference category

^*^values significant at *p* < 0.05

^**^values significant at *p* < 0.01

^***^values significant at *p* < 0.001

## Discussion

The purpose of this study was to compare the clinical recovery of adolescent SRC patients evaluated at a specialty pediatric concussion clinic within seven days since injury. Consistent with our hypotheses, the results showed that adolescents within the acute group were cleared three days faster than those in the sub-acute group, and six days faster than those in the post-acute group. Further, those evaluated within 48 h were approximately 4 times more likely to have normal recovery (recovery less than 21 days). Compared to other relevant variables associated with concussion recovery (initial symptom burden, sex, history of concussion, psychiatric diagnoses, neurodevelopmental diagnosis, Overall VOMS Change score, cognitive testing), early access to specialty concussion clinics, as measured by DSI group membership, was highly predictive of recovery time following PCSS total score.

Our results extend previous research, at a more granular level, and suggest that specialty concussion care, or a provider who is well-equipped to manage concussions, within 48 h, can significantly reduce recovery time compared to those who seek care beyond two days [23, 24, 40]. This is important as the literature has shown that established care with a provider who is familiar with the identification and rehabilitation of concussion is critical to recovery [2]. Historically, active management interventions such as prescribed physical activity or targeted rehabilitations were considered counterproductive or potentially detrimental [53]. For decades, the treatment of choice was complete physical and cognitive rest until the patient was asymptomatic [54]. It is only within the last two decades that research has shown the benefits of sub-symptom threshold physical exertion and vestibular rehabilitation, which too have consequently uncovered the possible deleterious effects of extended cognitive and physical rest [55–58]. Similarly, the literature has updated return-to-learn protocols showing that early return to school, with accommodations, is associated with lower symptoms at follow-up and faster recovery [58, 59]. This guidance and earlier care are important as optimizing clinical recovery can reduce consequences of post-concussive effects on daily functioning, falling behind in school, and emotional well-being [27–30, 60]. Indeed, prolonged concussive symptoms can increase psychological distress and compromise cognitive abilities [25, 61]. In addition, increased emotionality due to mechanisms like anxiety sensitivity can amplify physiological symptoms (and vice versa) leading to greater impairment than the initial injury [32, 62]. Protracted recovery has also been shown to increase healthcare costs and financial burden, which compound due to increased number of visits to hospitals and emergency departments [63]. Overall, the benefits of early concussion care include quicker access to education, tailored return-to-play and return-to-learn guidance, and follow-up maintenance appointments, which may reduce risk of protracted recovery.

This study is not without limitations. First, our main outcome variable, recovery time (i.e., time to clearance) is defined as the amount of time from initial appointment to follow-up appointment in which full medical clearance is received. The clinical judgement and expertise of the medical provider based on the patient’s presentation at the initial appointment, was used to determine the scheduled follow-up appointment to increase chances of medical clearance. There are myriad of factors that may affect recovery, which may take more or less time than expected. To increase internal consistency among providers, training regarding rehabilitation guidance, typical windows of recovery based on clinical presentation, and factors influencing recovery was implemented. If ambiguity arose based on a particular patient’s presentation, this was discussed with senior clinical staff to determine best course of action or whether inclusion within the database was appropriate. However, future research may decide to include a standard follow-up timeline to determine windows of recovery more accurately (e.g., check in every 7 days until medical clearance is achieved). Second, differences between clinical and statistical significance should be addressed. As clinical researchers, empirical evidence drives decision making in terms of inclusion or exclusion of predictor variables but may also differ depending on specialty. To address this, we included all factors in the GLM regression analysis that were relevant to the literature, regardless of statistical significance. We then identified the most significant predictors in our final analyses, the logistic regression, to ensure statistical and clinical relevance. Nevertheless, research regarding the ambiguity in clinical and statistical significance should be a point of future research inquiry in SRC. Third, greater inclusion of minoritized ethnic and racial backgrounds in this study are needed to better generalize findings. This underscores issues related to decreased or delayed access to specialty concussion clinics though more work is needed to fully understand these potential health disparities (non-private vs. private insurance) [64, 65]. Fourth, while there were no differences between DSI groups in terms of common variables that influence protracted recovery, we did not differentiate patients based on treatment recommendations (e.g., physical therapy, gradual return to activity guidance), which may have also influenced concussion recovery time. Evaluating recommended rehabilitation guidance in addition to comparing mechanism of injury, motor vehicle accidents, and military populations may be useful in understanding how earlier access to specialty concussion clinics influences recovery beyond SRC. The current study did not include participants who recovered spontaneously without medical guidance. Instead, we focused on individuals who sought specialty care based on concussion symptoms, especially during the acute phase of injury. Future research may include a comparison group of individuals who recover from their concussion naturally, without any specialized medical treatment, from those who seek care from a specialty concussion clinic. The findings may allow clinicians to better understand the factors that contribute to recovery based on medical guidance versus benefits that come from an individual’s daily activities or natural healing process. Additionally, our analysis focused on individuals who underwent their initial evaluation at a concussion clinic within seven days following their injury. Consequently, our dataset lacks information on outcomes for individuals who sought care after this seven-day period. It is important to note that existing studies have demonstrated that individuals receiving specialized concussion care beyond seven days post-injury are at an increased risk for prolonged recovery [23, 24]. Despite this, there is a potential for future research to refine our understanding by further stratifying the timeframe beyond the initial week post-injury (e.g., examining recovery outcomes for intervals such as 6–8 days, 9–12 days, 13–15 days post-injury) to delineate the impact of care timing more precisely on recovery trajectories. Lastly, we compared DSI groups with many similar demographics (sex, race, risks of protracted recovery, comorbidities) and found that early access was impactful in terms of recovery. Future studies should examine whether differences in access to clinic exist based on race and ethnicity, sex, comorbidities, type of insurance, or social determinants.

## Conclusions

A concussion may negatively affect quality of life, functionality, and worsening anxiety and depression, especially when time elapsed to care is considered [1, 30, 32, 42, 66]. Our results show that early access to care within a specialty concussion clinic within 48 h reduces recovery time by 3–6 days and increases the likely of normal recovery by 4 times compared to the post-acute group. Overall, receiving care as early as possible, awareness and education regarding concussive symptoms, and tailored rehabilitation protocols are vital in reducing recovery time.

## Data Availability

No datasets were generated or analysed during the current study.
